# Practical Points in Practice

**Published:** 1904-03

**Authors:** C. C. Noble


### PRACTICAL POINTS IN PRACTICE

By C. C. Noble, D.D.S.

Read before the Central Michigan Dental Association.

In presenting this paper I do not claim that the matter therein contained is essentially new, but I have found the methods set forth of great help to me, and I trust some of the points will be helpful to the members of this Society.

The first subject I will speak of is trichloracetic acid in the treatment of chronic abscess, more especially where we have a fistulous opening. I think we all have had more trouble with this class of cases than with most any other condition we are called upon to treat. My attention was called to this treatment at one of our Detroit Dental Society meetings. I am sorry I can not recall the name of the doctor who spoke of it. After two years' trial I can vouch for the efficacy of the treatment in the great majority of cases. In fistulous openings we have a condition that is impossible to cure unless we get rid of the dead tissue lining the opening, and also destroy the sac at the apex of the root.

Trichloracetic acid is a strong caustic and burns the tissues to a considerable depth, eating away the diseased tissue and sac, giving us a healthy surface that will heal very quickly.

My method of applyingit is to take a broach wound with a wisp of cotton, dipped in the acid, and pump the acid up through the opening on the gum. Care should be taken not to allow any of the acid to come in contact with the mucous membrane of the mouth, as it will burn and cause considerable discomfort. We do not get any of this trouble when careful, and the small amount coming through the opening on the gum does not trouble the patient, although it causes a whitening of the tissues immediately surrounding the opening.

After I have injected the acid I seal the tooth and dismiss the patient for two or three days; then remove the stopping and thoroughly wash out with peroxide of hydrogen, and repeat the treatment. Almost all cases will yield to two treatments. I would fill the root and tooth after the second treatment and expect, in a week at least, to find the fistula thoroughly healed.

Removal of the pulp with pressure anesthesia is sometimes rendered very tedious, by reason of the excessive hemorrhage. This can be overcome almost entirely if we will use adrenalin chloride with our cocain. Put a crystal of cocain in the cavity and cover with a very small pledget of cotton soaked in adrenalin chloride and press home with soft rubber. When the pulp is removed it will be found blanched almost white, and sometimes there will be absolutely no blood in the canal. I am indebted to Dr. Worboys for this hint.

In the removal of a pulp that has died and become soft and disintegrated, a wisp of cotton wound on a broach will be found very effectual in removing it. By gently twisting the broach with cotton we entangle the shreds of the pulp in the cotton and can remove the whole pulp at once.

A great deal has been written on root-canal filling, and each one has his favorite method and filling material, but I should like to call to your attention a preparation that I have found, after three years' trial, to be an excellent thing where we cannot remove all of the pulp from the cavity, and I claim this is a condition that the majority of us meet with very often. We read in our journals and hear men say at our meetings that they always get all the pulp out of a tooth. They may be honest in thinking they do, but just the same, I do not believe they do. It is one thing to demonstrate it on a tooth out of the mouth and an entirely different proposition before extraction. I have tried a great many preparations and have had the best results with a German preparation, Dr. Scheure's Formaldehyde Paste, sold by Gust. Scharmann, New York. After removing all the pulp possible, I force a little of the paste into the canal and then fill with gutta-percha. The paste is recommended as a root-canal filler and the whole canal may be filled with it, but I prefer to rely on gutta-percha for the bulk of the filling.

To those of you who are in the habit of making base plates for taking the bite for artificial dentures, I should like to recommend modeling compound in place of gutta-percha or wax. I know that many dentists use just a roll of wax without any base plate, but I am sure we will all admit that a more accurate bite can be obtained by using a base plate, and it is a difficult operation in many cases, under the most favorable circumstances. Modeling compound makes a very rigid base and is quickly and accurately adapted to the model, being much more easily worked than gutta-percha. It hardens very quickly, and the edges can be easily trimmed and smoothed with a knife, giving us a trial plate that fits the mouth perfectly and does not soften or change shape from pressure.

We are often called upon to repair a rubber plate that is so badly broken that it is a question whether a new plate would not be preferable, the question of expense to the patient, or time, being the only objection. The articulation being perfect, an entirely new denture can be made with very little trouble and almost as quickly as we can repair the old one, by the following method: Cement the pieces together as accurately as possible and insert in the lower half of the flask, keeping the plaster even with the edge of the rim of the plate. Oil the surface of the plaster, rubber and teeth, and build more plaster around the outside of the denture and up even with the occluding edges of the teeth; when this is sufficiently hardened, oil this surface, place the other half of the flask in position, fill with plaster and allow to harden. The flask will separate very easily, leaving the plaster rim around the outside of the teeth, which we next remove and place in the upper part of the flask. By heating the old denture the rubber can be easily removed, leaving the impression of the palatine portion of the mouth perfect. Remove the teeth from the old denture and place in the impressions in the upper part of the flask and proceed to pack the rubber as in any other case. When finshed, we have an entirely new denture with comparatively little work.

In bridge-work we often wish to confine the solder to one particular place and not allow it to flow over the adjacent parts. If you will paint the parts you wish to protect with yellow ochre water color, it will prevent the solder flowing over these parts; whiting will also work the same way, but when the heat is applied the whiting flakes off, which is not the case with the yellow ochre.

I have received a great many letters asking me for my experience with the Hurd Twentieth Century Gas Inhaler, and, as a recent inquiry was from a member of this Society, a few words on the subject may be of interest to the rest of you.

I have used the inhaler for over three years, and consider it the best method of administering gas I have ever seen. I have given it a great many times and have never had any bad results from it, although I have had failures, that is, patients that I could not get fully under the influence of the gas; but I do not think we should condemn the apparatus because it will not work every time, as I know of nothing we use, either as a medicine or in our operations, with which we do not sometimes have failures. The hood simply covers the nose, the mouth being propped open and the oral cavity exposed to full view all the time. When the patients are under the influence of the gas, we *keep them under* until our operation is done, and need have no fear of their coming to, as we do with the old method, as we have to remove the hood before we can do any work, and work against time. For the preparation of sensitive cavities, the grinding of a sensitive tooth, or the removal of a pulp it is unsurpassed; in fact, these operations are almost impossible with the old method. I have kept patients under from six to eight minutes, and believe there is no limit to the time. I think the amount of nitrous oxide used is about the same as with the Long apparatus, and also the time required to get a patient fully under; of course this varies, being from half a minute to a minute and a half. The patient inhales a mixture of the nitrous oxide and air, which our text-books say will prevent anesthesia. It certainly will with the Long apparatus, but works well in this way of administration, why, I have as yet been unable to ascertain. I very often, at the beginning of administration, hold the towel over the mouth, but I do not believe it necessary; I do it because I can not get over the feeling that they will go under quicker, although when I have not done so the results are perfectly satisfactory.

As soon as the patients become unconscious, the tongue drops back and compels them to breathe through the nose. We very seldom get the bluish cast to the skin and livid lips that you do with the old method, the patients hardly changing color at all—which is due, I think, to our not getting them into as profound an anesthesia as required with the old method, where it was *necessary* to get a deep anesthesia, as the administration ceased as soon as we began to operate, which is not the case with this apparatus.

### DISCUSSION.

DR. HATHAWAY: I have had trouble in forcing the mummifying paste into the fine canal, at least I am not sure that I get into the fine and crooked canals. I would like to know how Dr. Noble does this.

DR. NOBLE: I mix the paste to a consistency that will flow easily and introduce it into the canal with a suitable probe or broach and then force it with a broach on which cotton is wound so that it makes a piston. A ball of cotton will in some cases be sufficient if placed over the root opening and then pressed with a suitable blunt instrument, such as a burnisher.

DR. HINES: I have found that the tendency of porcelain facings to discolor when the backings are soldered to place may be prevented by painting the surface of the facing upon which the backing is placed with a thin solution of whiting in water, being careful, of course, not to use enough to interfere with a close adaptation of the backing.

DR. WORBOYS: I have experienced some trouble in making seamless crowns, because in drawing down the thimbles the crowns would be so thin that they would not wear long. I overcome this now by using No. 28 plate to start with, and using a size smaller punch than is usually employed until the last hole is reached. When the thimble is put through this hole twice, the first time with a punch one size too small and then follow with the punch fitted for this hole and No. 30 gauge plate. By this means the top of the cap is not thinned at all by stretching.

The discoloration of facings during soldering will usually not take place if the backing fits perfectly and the work is made clean before investment for soldering. I take a great deal of satisfaction in backing facings with gold and platinum plate, that is plate made by sweating gold onto platinum; it is gold on one side and platinum on the other. If I want a yellow effect in the facing I put the gold next the porcelain, if I wish a blue effect place the platinum next the facing. This backing can be used quite thin, and is readily adapted and will not burn off as easily as a gold backing. I think it a great advantage in bridge and some crown-work.

I think most dentists do not appreciate the strain put upon bridges in the molar and bicuspid regions of the mouth. A bridge made by uniting the edges of the backings with solder will not, as a rule, be sufficient to support the strain to which it will be subjected. It will either bend and draw the teeth out of occlusion and cause irritation or break and go to pieces. Wherever it is necessary and possible I place a stiff bar from one abutment to the other and solder the dummies securely to it.

Dr. Hoff: I was particularly interested in the method of repairing vulcanite plates as suggested by the essayist. This is a part of our work that it seems to me we do not give the attention it deserves. We repair any kind of an old rubber plate, it matters not how old or badly-fitting it may be, with little or no thought as to whether it is the best thing to do for our patients. A rubber plate that has been worn ten years ought not as a rule to be repaired, and one that has been vulcanized four or five times is not fit to put into a patient's mouth. The method here suggested of taking out all the old rubber and replacing it with new rubber appeals to me as a very proper and hygienic proceeding. I would go a step farther than the essayist and take a new impression and refit the plate. In this way you give the patient the best possible repair, and it can be done with little more trouble and expense than to make repairs in the ordinary way. If we had a flask with the middle ring in three sections it would make such a proceeding simple, especially for partial plates or where there were deep undercuts.

---

Porous Plates.—Porous plates are caused by over-heat; take more time in vulcanizing, and have your vulcanizer steam-tight. —*Dr. Gustavus North, Hints.*

---

Clean Plates.—Coat your models with liquid-silex just before closing flask; the plate will be smooth and clean when vulcanized.—*Dr. Gustavus North, Hints.*

---

Polishing Plates.—Rubber plates can be nicely polished with oil and fine plaster, by rubbing the plate briskly with finger or woolen cloth.—*Dr. Gustavus North, Hints.*

---

Cleaning Saliva Tubes.—When your saliva tube becomes cloudy from any cause place a few drops of acid in it. The acid will dissolve the solid matter which causes the cloudiness.—*Dr. A. W. Thornton, Dental Review.*

---

To Prevent Bruising Plate When Swaging.—In swaging metal plates, that you will not bruise your plate, get a rubber tip usually used on crutches. Put this on the large end of your horn mallet and proceed. —*Dr. D. E. Sheehan, Summary.*

---

The Plaster Bench.—A piece of plate glass about a foot wide and two feet long, set in the plaster bench near the waste drawer, presents a smooth surface on which to set models, and which is easily cleaned.—*Dr. A. W. Thornton, Brief.*

---

Rapid Repair.—For rapidly repairing a plate when one or two teeth are broken out, dovetail the plate back of the pins, set in the tooth and pack in a quick-setting amalgam. It will hold as well as a rubber patch.—*Dr. D. E. Sheehan, Summary.*

To Define Cavity of Decay.—Place rubber dam on the tooth and apply full-strength formaldehyde solution to the carious region; it will clearly define the extent of the softened tooth structure.—*Dr. F. W. Low, Cosmos.*

---

Zinc Dies.—To save time in making small zinc dies for special purposes the model can be partly contoured with wax, covered with tinfoil, and the mold made immediately.—*Dr. Mark G. McElhinney, Review.*

---

An Aid in Carrying Pumice.—When using felt cones or wheels in polishing rubber plates, gold crowns, etc., hold a piece of soap against the wet cone before applying the pumice. The soap on the cone prevents too much heat and will also carry the pumice.—*Dr. D. E. Sheehan, Summary.*

---

How to Prevent a Mouth-Mirror from Being Scratched by a Stone in Preparing Teeth for Crown Work.—Place a moistened microscopic cover glass upon the mouth-mirror. If the stone should mar it, it can easily be replaced, thus saving your mouth-mirror many a scratch.—*Exchange.*

---

To Repair a Hole in a Crown.—A small button of pure or coin gold hammered over a hole in the draw-plate, corresponding in size to the hole in the crown makes a convenient rivet which can be attached with solder, or, if a bridge is involved, can be riveted.—*Dr. Oliver Martin Review.*

---

Inserting Soft Cement.—When applying very soft cement over a pulp capping smear the tip of the instrument with phosphoric acid before allowing the instrument to come in contact with the mixed cement. This will prevent the adhesion of the cement to the instrument.—*Dr. Abraham M. Waas, Review.*

---

Soreness of Gingivae After Crown Setting.—If the soreness is persistent slip over the tooth a ring cut from rubber tubing. Remove carefully the next day and spray the parts with tepid water and you will in all probability find a minute nodule of cement. Remove and the gum will rapidly heal.—*Dental Review.*

---

Making Worn-Out Barbed Broaches Smooth.—Fold a small piece of fine emery paper, bringing the rough surfaces in contact, hold between the thumb and index finger of the left hand and work the broach back and forth between, rotating at the same time.—*Dr. F. J. T. Borchers, Review.*

---

A Soldering Hint.—When you solder your bridge, first of all have the investment hot. When using the blowpipe do not blow a strong blast, but blow gently enough to have a yellow streak in the flame from the pipe to the piece to be soldered. The yellow streak is essential. You can drag the solder with a piece of strengthening bar stuck in the end of a pine stick.—*Dr. D. E. Sheehan, Summary.*

---

Wax Sticks.—Get three or four glass tubes such as druggists use, about one-half inch in diameter, outside. Save your old base-plate wax and melt. Just before pouring wax, blow through the tube, the moisture will prevent it from sticking. Cork one end and pour. Set aside to cool, and when perfectly hard you can pull out a nice wax stick as smooth as though it had been turned.—*Dr. O. E. Hanford, Summary.*

---

Pulp Capping.—Carbonized cotton, like all carbon, is a poor conductor of heat and electricity, a thing to be considered in pulp capping. It can be thoroughly sterilized and used in all cavities where the walls are not so thin as to allow the color to appear through. Dip into the medicament, place carefully over the pulp and seal with temporary stopping—preferably formalin cement, which hardens rapidly, is a powerful antiseptic, and can be removed if necessary.—*Dr. A. Jessel, Dental Review.*

---

To Burnish Backing Over the Edges of Teeth.—In backing up facings for bridges and crowns with heavy plate, especially cuspids, it is sometimes hard to burnish the backing over the incisive edge. To facilitate matters split the backing in several places parallel with the long axis of the tooth to the line where the plate is to bend. It will then burnish over easily. Go over this surface once with a file and it will hug the tooth perfectly.—*Dr. D. E. Sheehan, Summary.*

---

Mending Broken Models.—In case of a tooth broken from a plaster or combination model, repair it by the use of cement, same as used for fillings. Moisten each fractured surface with the liquid, then apply thin cement, and press the fractured parts in to place; when sufficiently hardened it will be the strongest part of the model. —*Dr. Gustavus North, Hints.*

---

Painless Pulp Removal.—Equal parts of chloroform and carbolic acid. Using a French syringe (glass barrel, with glass piston, without needle, simply a canula), pack gutta-percha around the nozzle to prevent escape, and inject, forcing the piston down. The pulp can be immediately twisted out, blanched perfectly white, and insensible to pain.—*International Dental Journal.*

---

Cleaning the Cement Slab.—I notice from the last Journal that some dentists have trouble with the cement slab. I do not. After the "mix" is used, drop the slab in water. In working cement with plugger or broaches, dip in water and wipe off occasionally. Cement does not stick to instruments—a good thing to know, especially in root-filling.—*Dr. P. H. Callahan, Texas Journal.*

---

A Handy Method of Using Moldine.—Keep a piece of moldine of suitable size on a soft wood block, as this not only soon becomes saturated with the glycerine of the moldine, thus preventing a further drying out, but allows (when the die is to be of a carved cusp) a full view of all sides without disturbing the imbedded crown, or stooping over to see if you have imbedded the cusp far enough for your purpose.—*Exchange.*

---

Taking up Excess of Mercury.—Noticing that precipitated silver has been suggested for taking up the excess of mercury from an amalgam filling, I wish to suggest a method I have found effective: Squeeze very dry that portion of amalgam that is left after filling and hold it against the finished filling for a second, remove it, squeeze out and apply it again until all excess of mercury is removed.—*Dr. W. B. Garrett, Dental Era.*

---

Preparing Wax for the Laboratory.—Beeswax, resin and plaster of Paris, equal parts, makes a splendid wax for cusp carving. It is also a useful material for holding facings in position on the model in bridge work. Melt the wax, add resin, and while in the melted state add plaster of Paris, stirring until cool. If the result is found unsatisfactory melt again and add one or two of the three as required to produce the desired results.—*Dr. Homer Almon, Review.*

---

Sharpening Instruments.—Cut discs an inch and a half in diameter from emery paper, Nos. 00 and 1-2, and place in alternate layers to about half an inch in thickness, upon a disc of tin slightly smaller, and fasten on a screw mandril. On the disk of coarse paper bring the instrument to desired shape; tear off this disk and finish the sharpening on the finer grit. In this way a keen, even edge can be given to the instrument in a very short time.—*Dr. F. J. Patterson, Dental Review.*

---

Sterilizing the Hands.—The hands may be rendered practically sterile by the use of permanganate of potash, or, better still, a new preparation called sublamin, which is about as powerful as the corrosive sublimate, but does not irritate the hands. It dissolves well in water, can be used with soapsuds, penetrates deeper, and leaves the hands smooth. After the hands have touched infective material they should always be washed with sublamin.—*Dr. S. A. Hopkins, International Dental Journal.*

---

Diagnosis.—Heat a broad-faced amalgam instrument, take upon it a piece of gutta-percha, high grade preferred on account of its ability to retain the heat, hold it in the flame until nearly ready to burn, then touch the tooth, gently at first, and you will immediately determine the relative sensitivity of the pulps of the different teeth. If pulpitis is present, the pain will not immediately subside upon the removal of the hot gutta-percha.—*Dr. J. W. Beach, Forum.*

---

To Keep a Supply of Soft Wax.—Punch a hole the size of your Bunsen burner in the bottom of a seamless salve-box. Push this down to a point just above the air-holes in the burner and solder in place. Fill box with scrap wax, and when the burner is in use you will always have an abundance of soft wax. With this convenience you will save the time usually consumed in picking up and balancing a piece of hard wax on the end of your spatula in heating it.—*Dr. D. E. Sheehan, Summary.*

---

Restoring a Glazed Surface to Ground Porcelain.—Dissolve a small quantity of borax in distilled water and make a thin paste with Jenkins' low fusing body, the shade of the crown. Spread a thin film over the ground surface and fuse in the furnace. You will find that a beautiful surface has been obtained without injury to the porcelain if the piece is not overheated, which might occur if a high fusing porcelain was used. I have tried this several times with good results.—*Dr. C. J. Hadley, Review.*

---

The Age of Regulation.—If the permanent teeth erupt in malposition, treatment in the early stages is simplicity itself. The teeth are loosely surrounded by spongy bone and are easily moved; force is not needed; simply a gentle pressure to slowly guide the teeth to their proper position. As this is simply following out what nature is trying to do, there is little or no soreness in moving the teeth and not the slightest danger to the large, half-developed pulp of the developing tooth. Waiting always complicates the treatment. —*Dr. J. N. Macdowell, Dental Summary.*

---

The First Permanent Molar.—I do not always abandon the first molar because the pulp is exposed, for those pulps will bear more mutilation and recover more rapidly than those in the teeth of adults, so it is worth while to make the effort as wisely as our experience and judgment will allow. My observation on the removal of the pulps of thin teeth earlier that the twelfth year is against the operation. More to be feared than alveolar abscess is the deterioration of the matrix of the dentine that goes on slowly, so that in after life the teeth are not strong and will break down despite our best efforts.—*Dr. G. V. Black, Dental Digest.*

---

Pasteboard for Vacuums.—A good and economical vacuum material is pasteboard, a box such as rubber comes in will do. I find that this makes a better vacuum than metal, as there is no danger of slipping or the corners cutting through the plate. My way of using the board is this: When ready to wax up my plate I cut a vacuum of wax the size I want my board and put it in place, and then put my wax plate over it, then when ready pack my case, varnish with silex and put my paste-board vacuum in, the size of the wax one. Any one once using pasteboard will never use anything else.—*Clippings.*

---

Investment Material.—An investment material for bridge work, that will not check or break, even under rough treatment, may be made by taking long fiber asbestos (purchased from a dealer in plumber's supplies in the form of an asbestos rope), picking it to pieces with the fingers, and placing the fiber in the water, to which the plaster of paris is afterwards added. This investment dries out very quickly, does not change form and never requires to be wired or banded. It is inexpensive and one or two trials will enable any one to determine the proper proportions of water, asbestos fiber and plaster.—*Stomatologist.*

---

An Easy Way to Save Fine Gold Dust. —Where a crown or a piece of gold work is being dressed down with a stone in the handpiece, it is often unhandy to grind over some receptacle to catch the particles of gold. The difficulty may be obviated if you will provide yourself with a good-sized piece of wooly carpet for an apron. This allows one to grind in the handiest position, at the same time entangling the flying gold particles in the carpet. Keep the apron rolled up when not in use. This in time may be burned to secure this gold—as is done in our mints—or the particles may be dusted out into a paper and collected. The amount of gold thus saved will surprise you.—*Exchange.*

